# Well informed physician-patient communication in consultations on back pain – study protocol of the cluster randomized GAP trial

**DOI:** 10.1186/s12875-019-0925-8

**Published:** 2019-02-25

**Authors:** Sebastian Voigt-Radloff, Andrea C. Schöpf, Martin Boeker, Luca Frank, Erik Farin, Klaus Kaier, Mirjam Körner, Katharina Wollmann, Britta Lang, Joerg J. Meerpohl, Ralph Möhler, Wilhelm Niebling, Julia Serong, Renate Lange, Piet van der Keylen, Andy Maun

**Affiliations:** 1 Institute for Evidence in Medicine (for Cochrane Germany Foundation), Faculty of Medicine and Medical Center, University of Freiburg, Breisacher Str. 153, 79110 Freiburg, Germany; 2 Section of Health Care Research and Rehabilitation Research, Faculty of Medicine and Medical Center, University of Freiburg, Freiburg, Germany; 3 Medical Data Science, Institute of Medical Biometry and Statistics, Faculty of Medicine and Medical Center, University of Freiburg, Freiburg, Germany; 4 Friedrich-Alexander-University Erlangen-Nürnberg, Institute of General Practice, Erlangen, Germany; 5 Division Methods in Clinical Epidemiology, Institute of Medical Biometry and Statistics, Faculty of Medicine and Medical Center, University of Freiburg, Freiburg, Germany; 6 Medical Psychology and Medical Sociology, Medical Faculty, Albert-Ludwigs-University, Freiburg, Germany; 7 Cochrane Germany Foundation, Freiburg, Germany; 8 Clinical Trials Unit of the Medical Center, University of Freiburg, Freiburg, Germany; 9 School of Public Health, Bielefeld University, Bielefeld, Germany; 10 Division of General Practice, Medical Center – University of Freiburg, Faculty of Medicine, University of Freiburg, Freiburg, Germany; 11 Institute for Journalism, Technical University of Dortmund, Dortmund, Germany; 12 Bavarian State Association of Company Health Insurance Funds, Bavarian, Germany

**Keywords:** Lower back pain, Health communication, Internet portal, Primary health care, Evidence-based medicine

## Abstract

**Background:**

Back pain is one of the most frequent causes of health-related work absence. In Germany, more than 70% of adults suffer from at least one back pain episode per annum. It has strong impact on health care costs and patients’ quality of life. Patients increasingly seek health information on the internet. However, judging its trustworthiness is difficult. In addition, physicians who are being confronted with this type of information often experience it to complicate the physician-patient interaction. The GAP trial aims to develop, implement and evaluate an evidence-based, easy-to-understand and trustworthy internet information portal on lower back pain to be used by general practitioners and patients during and after the consultation. Effectiveness of GAP portal use compared to routine consultation on improving communication and informedness of both physicians and patients will be assessed. In addition, effects on health care costs and patients’ days of sick leave will be evaluated.

**Methods:**

We will conduct a prospective multi-centre, cluster-randomized parallel group trial including 1500 patients and 150 recruiting general practitioners. The intervention group will have access to the GAP portal. The portal will contain brief guides for patients and physicians on how to improve the consultation as well as information on epidemiology, aetiology, symptoms, benefits and harms of treatment options for acute, sub-acute and chronic lower back pain. The GAP portal will be designed to be user-friendly and present information on back pain tailored for either patients or physicians in form of brief fact sheets, educative videos, info-graphics, animations and glossaries. Physicians and patients will assess their informedness and the physician-patient communication in consultations at baseline and at two time points after the consultations under investigation. Days of sick leave and health care costs related to back pain will be compared between control and intervention group using routine data of company health insurance funds.

**Discussion:**

The GAP-trial intends to improve the communication between physicians and their patients and the informedness of both groups. If proven beneficial, the evidence-based and user-friendly portal will be made accessible for all patients and health professionals in back pain care. Inclusion of further indications might be implemented and evaluated in the long term.

**Trial registration:**

German Clinical Trials Register DRKS00014279 (registered 27th of April 2018).

**Electronic supplementary material:**

The online version of this article (10.1186/s12875-019-0925-8) contains supplementary material, which is available to authorized users.

## Background

The GAP trial will compare the communication of general practitioners and patients in consultations using an evidence-based, up-to-date, and independent internet portal in plain language with the communication in usual consultations without the use of the internet portal. The acronym GAP stands for the German words for “well informed physicians and patients”. In English it stands for **G**eneral practitioner **A**nd **P**atient communication. The study will evaluate the effects on physicians’ and patients’ perception of being well informed, the patients’ participation in the consultation, and the quality of the physician patient communication.

Both patients and physicians identified the lack of easy-to-understand and easy-to-use information resources as a barrier to well informed health decisions [1]. Barriers reported by physicians are (1) availability of scientific publications in English only, (2) complicated and expensive accessibility of e-journals and databases, and (3) lack of time to obtain the information, especially at the point of care and during decision-making [2].

The majority of globally generated evidence syntheses on effects of health care interventions are only available in English [3–5]. Accordingly, possible benefits and risks are often not sufficiently communicated at the point of care [6]. Patients may often barely understand information material, even when written in their native language. Consequently, they might not be able to informedly participate in medical consultations and shared decision-making [7–9].

In addition to face-to-face medical consultations, patients seek health information on the internet [10]. However, internet sources can hardly be appraised by patients [11–13]. Physicians sometimes experience internet information as irritating for the physician-patient relation and communication, thereby affecting the treatment outcome.

Back pain was chosen as medical condition of investigation because of its high burden of disease, its substantial impact on patients’ quality of life, and its relevance for public health [14]. The one-year prevalence account for more than 70% in Germany [15]. Chronic back pain is one of the most frequent causes for sick leave and early retirement, and related health care costs: Patients with back pain insured by company health insurance funds (BKK) were on sick leave due to back pain for 1700 days per 1000 insured persons and annum [16].

The optimal treatment strategy for back pain is controversial and approaches differ considerably between medical specialties, institutions and regions. The average cost for back pain treatment is 1322 € per patient and year, with 46% being direct costs for health care [17]. The German Council of medical experts reported over-, under- and misuse of care in back pain [18]. Adherence to clinical guidelines is low [19–21], and better coordination of back pain care may reduce the high costs of chronification [22].

In order to synthesize the evidence in the field of interest, we analysed Cochrane systematic reviews on generic and specific interventions to improve either medical consultation in general or health decisions in back pain.

• Dwamena and colleagues [23] reviewed 43 RCTs regarding the improvement of patient-centred medical consultations in primary care and concluded that training can increase physicians’ and nurses’ skills in patient-centred counselling. However, effects on patients’ health status, behaviour and satisfaction with consultation remained unclear. Evidence indicates that complex interventions addressing both patients and health professions might be more effective. Further research is necessary to confirm this hypothesis.

• A review on shared decision-making including 38 RCTs and one CCT revealed only 3 doublets with the review of Dwamena et al. and comes to congruent conclusions. Evidence quality is low but indicates that involving patients and health professionals may be more efficacious than addressing only one group [24].

• A review of 65 RCTs on patients, who were asked to give informed consent to surgery or other invasive interventions, showed that the use of written or audio-visual information material increased the patients’ knowledge about the planned interventions and reduced decisional conflicts [25]. The use of the information material showed no effects on generalized anxiety or anxiety with the consent process.

• Car and colleagues [26] assessed interventions for enhancing consumers’ online health literacy. In one RCT with HIV patients, internet health information classes improved (1) the self-efficacy for health information seeking, (2) health information evaluation skills, and (3) the number of times the patient discussed online information with a healthcare provider. In one CCT with healthy adults aged 50+, training improved the readiness to adopt the internet as tool for preventive health information. The review authors concluded that well-designed RCTs with various user-groups and 1-year-follow-up assessment are needed.

• A review of 24 RCTs on interactive applications for health communications in patients with chronic diseases revealed that knowledge, self-efficacy, social companionship, health behaviour and clinical outcomes were improved by interactive applications including an information portal combined with additional support either for decision-making, behaviour change or social support [27]. These findings, however, should be confirmed by well-designed RCTs.

• Engers et al. [28] reviewed 24 RCTs on individual education of patients with back pain. They found positive effects on return-to-work by intensive individual patient education in people with subacute back pain, but no effects of low frequent education and on long-term pain reduction. For chronic back pain, individual education was less effective for back pain-specific function when compared to more intensive interventions.

• A review on professional interventions for general practitioners on the management of musculoskeletal conditions analysed 10 trials on back pain [29]. Dissemination of and education on guidelines did hardly or not improve the generals practitioners’ adherence to guidelines. The combination with feedback on the total number of investigations or reminders attached to radiology reports reduced the number of investigation requests.

Summarizing these evidence syntheses, we conclude that (1) interventions for the improvement of shared decision-making and patient-centred communication should address both general practitioners and patients, (2) written or audio-visual material and interactive e-health applications may improve patients’ knowledge, decision-making and self-efficacy, (3) knowledge about successful use of internet sources for finding health information in general and in back pain specifically is limited, and (4) intensive education of patients and specific feedback for general practitioners is necessary in order to change patient behaviour or to reduce the number of diagnostic requests in back pain.

As evidence is still not optimally used for evidence-based decision-making at the point of care in primary care consultations, the GAP-trial intends to develop, implement and evaluate an easy-to-understand and easy-to-navigate health information internet portal for general practitioners and patients. Benefits of primary care consultations using the portal (intervention) and not using it (control: consultation as usual) will be compared in patients with back pain.

## Methods

The GAP trial uses a prospective multi-centre, cluster-randomized parallel group design in order to evaluate whether the use of the GAP portal on back pain is superior to routine care. The GAP project consists of three phases. (1) Within the pilot phase, the portal will be tested for usability by physicians and patients and adapted according to the pilot findings (Additional file 1: Development and piloting of the GAP portal). (2) The main trial (second phase) evaluates the effects on pre-defined outcomes in general practitioners and patients with back pain (Table 1). (3) Within the third phase, the potential benefit of web-based information for health problems other than back pain will be analysed (Additional file 2: Expanding of the GAP portal).

**Table 1:** PICO question of the GAP trial

Research Question	What are the effects of using the back pain internet portal compared to standard care in patients with back pain?
Population	150 general practitioners and 1500 back pain patients in Northern Bavaria
Intervention	Using the portal during (physician) and after (patient) the consultation.
Comparison	Routine consultation (no use of the portal)
Outcomes	1. Physicians‘and patients’ informedness 2. Physician-patient-communication 3. Patients‘health literacy and self-efficacy expectation 4. Days of sick leave 5. Back pain related health costs

*Hypothesis on primary outcomes:* (1) After the consultation, the informedness and the quality of the physician-patient communication are rated significantly higher by general practitioners and patients who use the portal (intervention group, IG) than by those who do not (control group, CG, consultation as usual).

*Hypotheses on secondary outcomes:* (2) 3 weeks after the consultation, the patients’ informedness, health literacy and self-efficacy are significantly higher among those who use the portal than among those who do not. (3) 3 months after consultation, the number of days of sick leave as well as the utilization of health system resources is significantly lower among patients who use the portal than among those who do not.

*Design:* Prospective cluster RCT with pre-, post- and one follow-up-measurement and process evaluation.

### Participants and recruitment

Together with the *Bavarian Association of Family Physicians and General Practitioners,* the *Institute of General Practice at the University Medical Center Erlangen* (UHE) will recruit a total of 150 general practitioners (GP) from practices in Northern Bavaria. Two-thirds of the practices are randomized to the IG and one third to the CG. If a GP practice expresses interest after being invited to participate in the study, it receives information concerning the study, regulations for enrolling in the intervention program, and participation documents for the GP practice and the patients. After written consent to study participation and enrolment in the intervention program, the practice is formally included in the GAP trial. The UHE passes on registration data to the *Bavarian State Association of company health insurance fund*, which is responsible for the cooperation with the participating company health insurance funds. Over five quarters (July 2018 to September 2019), the GP practices are to recruit a total of 1000 patients for the IG and 500 for the CG. With an average share of at least 20% BKK insured persons, 900 patients per practice license in Bavaria and a back pain prevalence of almost 30%, the resulting recruitment potential is 50 to 60 patients per physician. The fact that physicians receive reimbursement for recruitment and patients receive a book voucher will facilitate sufficient recruitment.

#### Eligibility criteria for patients

Eligible are persons aged 18 years or older, insured by a company health insurance fund, presenting with back pain symptoms in a participating GP practice and providing written informed consent. Patients with insufficient German language skills to fill in the study questionnaires are excluded from the study.

#### Termination criteria

No termination criteria are defined for the overall study. The withdrawal of written consent is the termination criteria for the individual patient respectively physician. In the event of a withdrawal, deletion from the assignment list is ensured and the related questionnaires are immediately destroyed and erased from evaluation. Physicians and patients are informed that already processed data will still be used anonymously.

### Interventions and procedures

#### Description of the portal

Evidence-based information on treatment options for back pain is comprehensibly presented and made available as a login-required, password-protected online portal for participating physicians and patients. The portal focuses on frequently occurring issues in general practice related to back pain. It contains descriptive material, links to further information and suggestions for well-informed shared decision-making, self-responsible training and preventive lifestyle changes.

During consultation, the physician can show images, animations, infographics and videos from the portal to the patient, discuss the material, and provide the patient with a concise selection of information as a printout. To ease selection, the portal will contain four pre-structured information packages for common back pain problems:

1. Acute non-specific lower back pain.
2. Sub-acute non-specific lower back pain.
3. Chronic non-specific lower back pain.
4. Brief information on certain diseases causing specific lower back pain.

The information packages are designed to be highly accessible for physicians and patients and contain clearly navigable information on epidemiology, causes, symptoms, harms and benefits of treatment options and on the reliability of the given information. The evidence of recommended and not-recommended treatment options (such as physiotherapy, pain medication or bed rest) for the most common conditions are outlined including risks and side effects. Each package is designed to motivate navigation and quick understanding, and includes references to further information for physicians and patients. In addition, the patient receives multimedia instructions for self-management strategies and training. Furthermore, the portal provides two customized reference books for physicians and patients in order to enable the search for information by keywords.

For the physician, the portal includes user instructions and a brief guide on how to structure the consultation. The guide recommends the following established consultation pattern: (1) patient’s concerns and expectations, (2) physical examination, (3) working hypothesis or diagnosis, (4) therapy proposal and shared decision-making. For the patient, the portal includes explanations and guiding questions to prepare for a well-informed physician patient communication.

#### Procedures for physicians

Physicians in IC and CG fill out the primary outcome questionnaires at the following time points: before intervention (before accessing the portal (t0), directly after recruiting the last patient (t1), and 3 months after recruiting the last patient (t2). Data from the CG is gathered concurrently. Physicians in the IG are interviewed online, physicians in the CG via an online survey or, where appropriate, paper-pencil questionnaires (hybrid survey). Structural features of practices are collected once in an initial questionnaire in both IG and CG. After the intervention (t1) in the IG, usage, usability and accordance of information and information needs are surveyed. To test for enduring use, the usage of the platform is again measured at t2. Physicians in the CG continue to perform their usual consultation routines. The participating physicians of the IG and CG are given an incentive for study participation by the UHE at the end of the survey period. The incentive is calculated according to the number of recruited patients per physician (34 € per participating patient in IG and CG). Additionally, the physicians in the IG receive 40 € once per patient for the first consultation using the portal. The study protocol does not determine any limitations or specifications for further consultations or the care of the patients. Physicians of the IG are asked to use the portal during the consultation according to the instructions and the consultation guidance. To ensure appropriate usage of the portal, the UHE sets up a telephone hotline for the physicians in case of difficulties or questions. During the initial phase, the institute contacts each IG physician at least once proactively, and offers support. This proactive contact is made step by step. First, the physicians are contacted by email and asked whether support is needed. If there is need for support, telephone contact is offered and, if necessary, a personal visit is made. As an adherence indicator the frequency of portal usage per physician is measured via web analytics. 25 IG physicians are randomly selected and interviewed by the Institute of Medical Psychology and Medical Sociology (MPS) after inclusion of the last patient in semi-structured telephone interviews on portal acceptance, quality and practicability (implementation, supportive factors and barriers) and the perception of their own communication behaviour as well as the subjective evaluation of the consultation guidance.

#### Procedures for patients

**Fig. 1:**
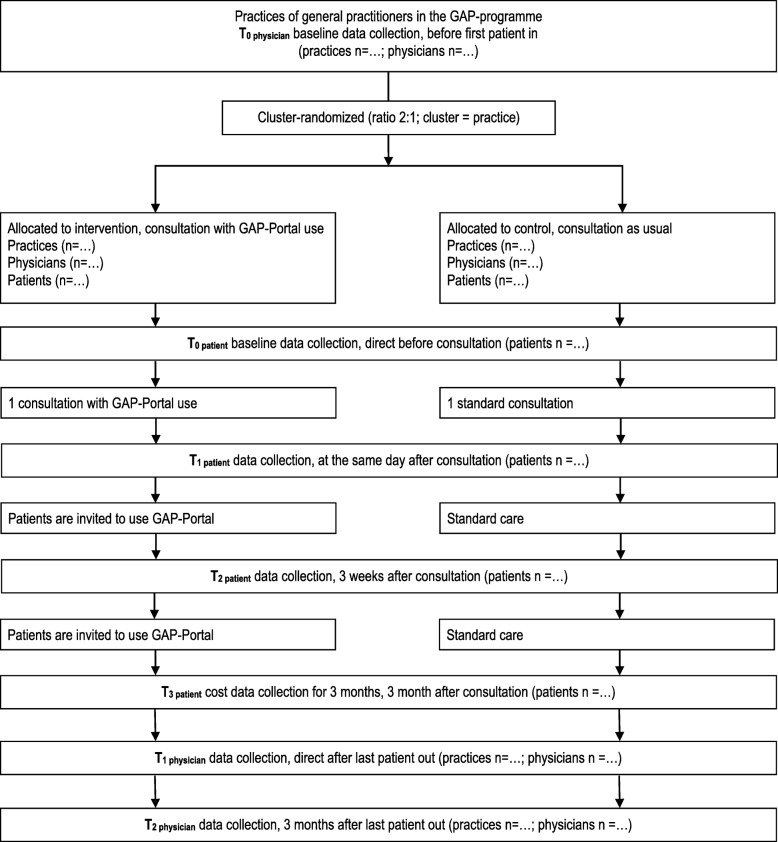
GAP Flow Diagram. Numbers and reasons of discontinuation of intervention and of lost to follow up will be given for each group

In both IG und CG all patients with back pain receive brief study information at their first consultation and are invited to participate in the study. If interest is expressed, the physicians will inform the patients and enrol them in the study. Each enrolled patient will receive a patient number consisting of a three-digit practice number, a two-digit physician number and a two-digit patient number (e.g. 543/09/01). This number matches the number of their questionnaire. Each physician receives questionnaire packages for 10 patients including: envelope with the t0 and t1 questionnaires and the log-in name for the portal (if patient is in IG). The packages are prepared by the Section of Health Care Research and Rehabilitation Research (SEVERA) on the basis of a pseudonymised list of all participating practices. After enrolling a patient, the medical practice immediately contacts the UHE and forwards the questionnaire number (patient number), contact information, written consent for study participation and permission for data usage to the UHE. Written consent must be received by the UHE in order to allow the UHE to send the t2 questionnaire along with a book voucher 3 weeks after consultation. Patients in IG and CG complete questionnaires on informedness, physician-patient communication, health literacy and self-efficacy at three measurement points: in the doctor’s office before (t0) and after (t1) consultation and 3 weeks later by post (t2). Patients in the CG receive consultation and care as usual not limited or influenced by this study. For patients in the IG the physician uses the explanatory material of the back pain portal, selects information and sends it as a printout or by email. The doctor encourages the patients to study the provided material, to access the online portal and to implement the given recommendations. With the log-in name received in the practice, patients in the IG can access the portal after their consultation. Usage frequency of the portal per patient is checked via web analytics and serves as indicator for intervention adherence. There are no other restrictions or requirements by the study protocol for additional care of the patients in the IG. Further consultations are covered by the health care system. Doctors and patients of the IG are free to continue using the portal during the study. Compensation for using the portal in consultation can only be charged once per patient. 50 randomly selected patients from the IG will be interviewed by SEVERA after completion of the postal survey (t2) in semi-structured 45- to 60-min telephone interviews on acceptance, quality and practicability of the portal as well as satisfaction with the counselling process and the perception of their own and the physician’s communication. Numbers and reasons of discontinuation of intervention and of lost to follow up will be given (Fig. 1).

### Outcomes

*Primary outcomes,* which are assessed by questionnaires (survey data), are the quality of the patient-physician communication and patient informedness (Table 2). It is analysed whether values in the IG differ significantly from the CG.

**Table 2:** Primary outcomes, instruments and measurement points

	t_0-physician_	t_0-patient_	t_1-patient_	t_2-patient_	t_1-physician_	t_2-physician_
Quality of the patient-physician communication (patient)						
Shared Decision-Making Questionnaire (SDM-Q-9) [38]			X			
Questionnaire on the communication behaviour of physicians (KOVA) [39]			X			
Scale “Satisfaction” of the P.A.INT-questionnaire [40–43]			X			
Quality of the patient-physician communication (physician)						
Scale “Effective and open communication” of the KOVA [39]^a,b^	X				X	X
Scales “Empathy”, “Openness and coherence”, “Positive regard and appreciation”, “Contact barriers” and “Satisfaction” of the P.A.INT-questionnaire [40–43]^a^	X				X	X
One Item of the Man-Son-Hing scale. [44]^a^	X				X	X
Shared Decision-Making Questionnaire (SDM-Q-Doc) [45, 46]^a^	X				X	X
Informedness (patient)						
Self-reported knowledge questionnaire^c^		X		X		
Self-reported knowledge global question^c^			X			
Perceived informedness questionnaire^c^				X		
Perceived informedness global question^c^			X			

^a^Instruments will be adapted so that physicians assess their communication behaviour across consultations

^b^Instrument will be adapted for assessing the communication from physician’s perspective

^c^These instruments will be developed for the project. The questionnaires will be checked for understandability and acceptance in a cognitive pretest using one-to-one interviews with eight patients having experienced back pain [47]

*Secondary outcomes* are assessed by questionnaires (survey data) and routine data from the insurance companies (Table 3). Secondary patient outcomes are self-reported self-efficacy regarding their own communication behaviour, health literacy, pain intensity, and days of sick leave and back pain related costs for the health care system. Since t0 and t1 of the patient survey are close together, outcomes of informedness, self-efficacy, and health literacy at t1 are only assessed via global questions. Secondary outcomes for physicians in the IG are the concordance of provided and needed information, the usability of the platform and their actual use of it. Correlation of physician and patient data is possible, as the used items and questionnaires are similar. Differences between CG and IG are additionally analysed regarding the secondary outcomes. Table 3 also contains additionally assessed variables.

**Table 3:** Secondary outcomes and control variables

	t_0-physician_	t_0-patient_	t_1-patient_	t_2-patient_	t_3-patient_	t_1-physician_	t_2-physician_
Secondary outcomes (survey data)							
Self-reported self-efficacy (patient)							
Perceived Efficacy in Patient-Physician Interactions (PEPPI)-Questionnaire [48]		X		X			
One Item of Perceived Efficacy in Patient-Physician Interactions (PEPPI)-Questionnaire [48]			X				
Health Literacy (patient)							
Health Literacy Questionnaire (HLQ) [31, 49]		X		X			
Health literacy global questions			X				
Pain intensity (patient)							
Visual analog scale (VAS) [50]		X		X			
Accordance of provided and needed information (physician)							
Domains “Adequacy of information” and “Usability of information” of the Decision Attitude Scale^a,b^ [51]						X	
Self-developed item whether content fits expectations						X	
Usability (physician)							
System Usability Scale (SUS)^a,b^ [52]						X	
Self-developed items on usability						X	
Use of portal (physician)							
Self-developed items on actual use of portal^b^						X	X
Secondary outcomes (routine data)							
Patient’s days of sick leave					X		
Patient’s back pain related costs for the health care system					X		
Control variables (survey data)							
Patient							
Back Belief Questionnaire [53]		X					
Sociodemographic data		X	X	X			
Internet Use		X					
Medical data		X		X			
Physician							
Sociodemographic data	X						
Structural features of the practice (kind of practice, patients per quarter, number of employees, location and region)	X						
Control variable (web analytics)							
Portal use by patients		Throughout the intervention phase (after consultation)					
Portal use by physicians	Throughout the intervention phase						

^a^Instrument will be adapted so that the physicians assess the portal

^b^only intervention group

#### Sample size calculation

A priori calculation of the sample size to test the IG’s superiority to the CG in primary outcomes (comparison of adjusted mean values in two groups) was performed with the software *“Power and Precision*”. We used an effect size of 0.30, power of 0.80 and a Bonferroni adjustment (5% / 2 = 2.5%) due to the multiple testing of two primary outcomes. The design effect due to clustering of the practices [30] is DE = 1 + 0.01 × (10–1) = 1.09 with an assumed intra-cluster correlation of 0.01 [31] and a number of cases per cluster of *N* = 10. With a 2:1 distribution in the IG and CG, a sample size of *N* = 320 (IG) and *N* = 160 (CG) without design effect and *N* = 349 (IG) and *N* = 175 (CG) with design effect was calculated. Due to an expected dropout of approximately 35% a sample size of *N* = 537 (IG) and *N* = 270 (CG) is necessary. However, health economics evaluation including a budget impact analysis requires a relatively large variance of cost values (150% standard deviation). Based on a power of 0.80, an alpha level of 5% and an effect size of 30%, the number of cases is *N* = 590 (IG) and *N* = 295 (CG) without design effect and *N* = 643 (IG) and *N* = 322 (CG) with design effect. Given the assumed dropout of approximately 35%, this results in the desired recruitment target of *N* = 1.000 (IG) and *N* = 500 (CG).

### Generation and concealment of allocation sequence and implementation of allocation

The participating practices are allocated in a 2:1 ratio of IG to CG. The allocation will be stratified by number of participating physicians within a practice (1 physician vs. 2–4 physicians vs. more than 4 physicians). The allocation sequence will be computer generated and concealed until time of allocation. The UHE will recruit the practices, while the MPS will generate the allocation sequence. The UHE will send a list with the research numbers of the practices and the stratification characteristics to the MPS which adds the information about group allocation according to the randomly generated sequence.

The UHE will inform the practices about their allocation. This procedure ensures that neither the recruiting nor the analysing institutes can influence that a particular practice will be allocated to either IG or CG.

### Blinding/masking

Due to the nature of the intervention, participants (physicians, patients) will be aware of their allocation status and cannot be blinded. Also, the assessment of the evaluators cannot be blinded, since the number of cases will differ between IG and CG, questionnaires for the IG will contain additional variables, and it is possible that further questions regarding the routine data of the health insurance arise.

### Data collection methods

In addition to interviews and the health insurance routine data, the intervention is mainly assessed using questionnaires completed by patients and physicians (Additional file 3: Characteristics of survey instruments). The practices will receive a detailed instruction manual in order to ensure standardised collection of data.

### Data management

A seven-digit patient research ID (three-digit number for the practice, two-digit number for the attended physician and a consecutive two-digit number per patient attending the physician) is allocated to each participating patient. A database with these research IDs and the associated names and contact details of the patients (participation list) is created. Due to the research IDs the questionnaires of the three measurement points can be matched. Further, the research IDs of the questionnaires are definite relatable to the log-in names of the portal (user data) and the research IDs used for the routine data of the health insurance. The analysing institutions keep a record about the return of the patient questionnaire in an Excel-Sheet. The ID of returning questionnaires will be reconciled with the ID of cases in the SPSS data files.

Codebooks will be prepared for the questionnaires. Scanning is used for the data entry of the patient questionnaires. The team member entering the data can immediately correct data which was incorrectly inserted by the computer system. The data of 10% randomly selected questionnaires will be double-checked. If more than 5 ‰ of the data entries are incorrect, all data will be entered twice. The patient questionnaires will be checked for plausibility of age and gender. The checks include that the gender is reported identically across all three measurement points and that there is only 1 year of difference between the measurement points t0 and t2. For all items there will be a range check of data values. As the physician questionnaires are completed primarily online, there will be no double entry of data but there will be plausibility checks comparable to the patient questionnaires.

### Statistical methods

#### Analysis of patients‘questionnaire data

Based on the analysis of the missing outcome data, simple or multiple imputation may be considered. Dropout analysis will be performed using Chi-Squared tests for nominal variables, Mann–Whitney U tests for ordinal variables and t-tests for interval variables. The significance level is set at *p* = 0.05. Special attention is paid to the hierarchical data structure of the questionnaire data. Due to the cluster randomisation, patients’ responses cannot be presumed as independent, as the patients are nested within practices. Because of this, separate two-level (patient – practice) multilevel linear models for both measurement points (t1 and t2) will be calculated. Treatment allocation (IG vs. CG) will be used as predictor variable in the multilevel linear models. The fit of the multilevel linear models are tested using amongst others the Akaike’s information criterion and Schwarz’s Bayesian criterion. Despite the randomisation a propensity score adjustment is considered in case of a potential imbalanced allocation [32]. Intraclass correlations are calculated to assess the cluster (practice) dependency of the responses.

#### Analysis of physicians‘questionnaire data

The data analysis is primarily exploratory. The primary outcomes will be analysed using inferential statistics methods. IG and CG will be compared across the three measurement points. Statistically adjusted comparisons of means between IG and CG as well as across the three measurement points (using propensity scores) will be conducted. The characteristics of the practices and the user data of the online platform will be used as control variables. Before the calculations, the missing data processes, singular missing data and the drop-out of patients will be analysed. Recommendations regarding the handling of missing data in order to correct information biases will be considered [33]. If data is missing completely at random or missing at random, missing data will be imputed using the expectations-maximation algorithm. Moreover, there will be sensitivity analyses to assess the influence of missing data processes.

#### Analysis of the routine data

The health economic analysis will be conducted in two steps. First, the financial effect of the intervention from the health insurances’ perspective will be calculated using a Budget-Impact-analysis. This analysis considers the back-pain related in-and outpatient costs for the health care system and the costs related to days of sick leave, where available. In an initial sensitivity analysis the reported days of sick leave are assessed in an alternative way using the human capital method to prevent potential inconsistencies. Depending on the data security regulations (see data security manual), the results of the Budget-Impact-analysis will then be linked to the outcomes of informedness using an exploratory cost-effectiveness analysis. The aim is to assess two cost-effectiveness ratios: additional costs per unit of ‘patients’ self-reported knowledge’ and additional costs per unit of ‘patients’ perceived informedness’. For the cost-effectiveness analysis confidence intervals will be calculated using the Fieller-theorem [34].

#### Process evaluation

An additional process evaluation comprises interviews with a sub-sample of patients and physicians and the analysis of the user data of the online platform using Web-Analytics. The interviews will be recorded and transcribed. The transcripts will be analysed using a framework analytical approach according to Ritchie and Spencer [35], Gale et al. [36] und Parkinson et al. [37]. The user data will be matched to the questionnaire data.

### Data monitoring

Participation in this study carries minimal to no risks in addition to those associated with standard care. Therefore, there is no Data Monitoring Committee. The quality of the study implementation and progress will be monitored by the leader of the consortium who is in continuous exchange with all consortium members. There are no interim analyses planned.

### Adverse events

All members of the project team forward reports about adverse events to the leader of the consortium, who inserts all reports into a log file.

### Auditing

There will be no independent audits.

### Protocol amendments

All substantial modifications to the protocol before or after the start of the recruitment concerning the inclusion and exclusion criteria, the conduct of the intervention or the evaluation will be documented and reported to the ethics committee and in the main publication according to the CONSORT statement.

### Consent or assent

The UHE will obtain written informed consent from the physicians and practices participating in the main study, while physicians of the participating practices inform patients about the study and obtain their written informed consent.

### Confidentiality

All personal data (consent forms, participation lists, audio recordings) will be kept in locked cabinets or will be password protected. In order to ensure data security, there will be 5 research IDs for the evaluation of the portal. Only the UHE will have a list containing the assignment of all 5 research IDs and names and contact details of the participants (physicians/patients). The UHE will not have access to any research data. The analysing institutions (MDS: Medical Data Science; MPS: Medical Psychology and Medical Sociology; and SEVERA have access to the pseudonymised questionnaire data (research ID 1) and the user data of the portal (research ID 2/log-in name). The Division Methods in Clinical Epidemiology of the Institute of Medical Biometry and Statistics (MICLEP) receives the routine data in addition (with research ID 3). The UHE sends the insurance numbers of the participating patients with research ID 3 to the *Bavarian State Association of company health insurance funds* which forwards the routine data with the research ID 3 but without the insurance number to MICLEP. The analysing institutions receive a list with the matched research IDs 1 and 2 respectively 1, 2 and 3. This procedure ensures that the personal data and research data are kept separate and the analysing institutes have no access to the personal data.

For organizational reasons (establishing of contacts) the analysing institutions (SEVERA, MPS) must have access to the contact details of patients (with research ID 4) and physicians (with research ID 5) for the interviews. The contact details will be deleted immediately after the interview phase. During the online survey no personal details such as names or contact details will be collected. For this project, a detailed data security manual was prepared, which is agreed with the data protection officials of the University Medical Center of Freiburg, the *Bavarian State Association of company health insurance funds*, the University Hospital of Erlangen and the Technical University of Dortmund.

### Access to data

For evaluating the portal, the analysing institutions have access to the pseudonymised questionnaire and user data; MICLEP also to the pseudonymised routine data. The Institute for Evidence in Medicine (for Cochrane Germany Foundation), the Division of General Practice in Freiburg and the Institute for Journalism in Dortmund will only receive anonymized questionnaire data. No research data will be forwarded to the UHE and the *Bavarian State Association of company health insurance funds*. According to the license agreements of the HLQ, there is the possibility that anonymized data of the HLQ and some additional sociodemographic and medical variables might be forwarded to the authors of the questionnaire in order to build a global data base in the future. The audio recordings of a research phase are only accessible to team members of the institutions responsible for the data collection. Team members of institutions which are participating in the analysis of a particular research phase have access to the anonymized transcripts of the recordings. The routine data can only be accessed by the team members of MICLEP. Access rights are explained in detail in the data security manual.

### Ancillary and post-trial care

As the risk of harm for participating physicians and patients is not increased by study participation, no additional insurances or ancillary care are planned. All patients receive standard care according to up-to-date guidelines during and after the study.

### Publication plan

The GAP trial consortium plans to publish and communicate trial design, procedures, context issues and results as follows:

1. Publication of the full trial protocol (the present paper).
2. Trial registration at the German Clinical Trials Register connected with the WHO register.
3. Systematic reviews on online information activities and needs of consumers and health professionals.
4. Publication of pilot results.
5. Publication of complete trial results on all outcomes described in the trial protocol, no matter if hypotheses will be confirmed or not.
6. Publication of results of the process evaluation including possible explanations why the intervention did or did not work.
7. If the intervention was successful, key points would be published on how the intervention can be implemented in routine health care and how it can be expanded to health care fields other than back pain care.
8. We will proactively inform relevant Cochrane Review Authors and Guideline Authors to consider our trial results when updating their reviews or guidelines.
9. We will present summaries of our results and insights on scientific symposia, on congresses for consumer groups and on public and social media.

The GAP trial consortium agreed on a common publication plan deciding on leading and co-authorship for each publication by discussion and consensus. If needed, we will ask English native speakers with translation expertise for support to provide manuscripts in proper English. Support from professional writers will not be used.

## Discussion

Our GAP-trial addresses the important issue of evidence transfer to the point of care and shared decision-making. If the study shows advantages of GAP-Portal usage, health literacy and decision quality could be improved by making the portal accessible to the public. Because the portal is designed together with users and to be used without any introductory seminar, we anticipate that it could be easily adopted by patients and health professionals in clinical practice. If the GAP portal demonstrated positive effects on informedness, communication and health outcomes, inclusion of further indications might be implemented and evaluated in the long term.

## Additional files

Additional file 1: Development and piloting of the GAP portal. (DOCX 65 kb)

Additional file 2: Expanding of the GAP-portal. (DOCX 301 kb)

Additional file 3: Characteristics of survey instruments. (DOCX 30 kb)

## Abbreviations

BKK: company health insurance funds
CG: control group
GAP: stands for the German words for “well informed physicians and patients”
GP: general practitioner
IG: intervention group
MDS: Medical Data Science
MICLEP: Division Methods in Clinical Epidemiology
MPS: Medical Psychology and Medical Sociology
RCT: Randomsed controlled trial
SEVERA: Section of Health Care Research and Rehabilitation Research
t0–4: time points of measurement
UHE: Institute of General Practice at the University Medical Center Erlangen
