# Supplementary material for: Well informed physician-patient communication in consultations on back pain – study protocol of the cluster randomized GAP trial
Source: BMC Fam Pract. 2019 Feb 25;20:33. doi: 10.1186/s12875-019-0925-8 (PMC6388488; doi:10.1186/s12875-019-0925-8)
Supplement: Supplementary file 1 — Development and piloting of the GAP portal. (DOCX 65 kb) [file 12875_2019_925_MOESM1_ESM.docx]

**GAP trial, additional file 1: Development and piloting of the GAP portal**

**Establishing the roles and main tasks of the development group**

| **Cooperation partners (professions)** | **Role** |
| --- | --- |
| Institute for Evidence in Medicine (for Cochrane Germany Foundation)  (researcher, physiotherapist, digital media designer) | Literature review, content preparation,  digital media production |
| Division of General Practice  (general practitioner, researcher) | User perspectives, content review |
| Web design company Tomes GmbH  (physician, web designer) | Design, branding, implementation |

Interprofessional subgroups work closely together on tasks and initially have weekly team meetings

**Defining the user groups and use cases (Division of General Practice & web design company)**

User groups:

| General practitioners (GP) | Patients |
| --- | --- |

Three consecutive use cases were identified

| 1) GP searches through evidence based guidelines and information on low back pain. |
| --- |
| 2) GP explains findings/therapies to the patient during consultation. |
| 3) Patient searches through explanations/advice at home after the consultation. |

**Result: two interconnected websites – for GPs and for patients. Corresponding content in professional
and plain language is produced and displayed on the website according to the use case**

|  | Use case 1 | Use case 2 | Use case 3 |
| --- | --- | --- | --- |
| User group / case | GP retrieves info | GP informs patient | Patient retrieves info |
| Language | professional | plain | plain |
| Dominant Information format | expandable texts, visual expandable guideline | short lists, illustrations and 3D model | videos for explanation and exercises, pictures, short texts |

Textual content, navigation elements, visual expandable guideline, illustrations, 3D model and videos are developed and reviewed in small inter-professional subgroups. Additional professionals are consulted (e. g. video production). Feedback on produced materials and navigation is retrieved from GPs in rapid development cycles.

**Structured feedback from the pre-tests is used to restructure
the navigation on the website and to refine the design and content.**

Feedback on newer versions is retrieved from GPs and patients in rapid development cycles.

**Eye-tracking pre-test: GPs/physicians and patients**

The pre-final GAP-Portal is to be tested in an eye-tracking experiment by communication scientists from the Institute for Journalism at the Technical University Dortmund. Ten to twelve GPs with varying degrees of experience and ages from the Freiburg region will be asked to test the pre-final version of the back pain portal for physicians. Similarly, thirteen to fifteen patients with back pain (self-assessment) will be recruited at a rehabilitation hospital in Dortmund for the eye-tracking experiment on the portal’s usability for patients.

During the use of the portal (approx. 15 minutes) navigation is observed by the researcher and eye movements of the participant are captured with an eye-tracking camera system (actual navigation). The stimuli can vary between individual screenshots or free use of the entire portal. The participants will be asked to perform search tasks and to compare different designs. They will be encouraged to comment on their actions and impressions during the experiment. Recordings of the participants’ comments will be considered in the analysis and interpretation of the eye-tracking data. Semi-structured interviews based on key questions will follow immediately and ask for the perceived understandability and usability of the portal (perceived understandability and usability). The interviews will provide more detailed information concerning specific problems of the pre-final version as well as user expectations and needs. Results from the pilot testing will thus serve as an input for the development and optimization of the portal for physicians as well as for patients.

**Eye-tracking pre-test: 12 GPs/physicians and 12 patients**

**Structured feedback from the pre-tests is used to restructure the navigation on the website and to refine the design and content.**

Feedback on newer versions of the is retrieved from GPs and patients in rapid development cycles.

**Structured feedback from the pre-tests is used to restructure the navigation on the website and to refine the design and content.**

Feedback on newer versions of the is retrieved from GPs and patients in rapid development cycles.

**Eye-tracking pre-test: 12 GPs/physicians and 12 patients**
