# Supplementary material for: Well informed physician-patient communication in consultations on back pain – study protocol of the cluster randomized GAP trial
Source: BMC Fam Pract. 2019 Feb 25;20:33. doi: 10.1186/s12875-019-0925-8 (PMC6388488; doi:10.1186/s12875-019-0925-8)
Supplement: Supplementary file 2 — Expanding of the GAP-portal. (DOCX 301 kb) [file 12875_2019_925_MOESM2_ESM.docx]

**GAP trial, additional file 2: Expanding of the GAP portal**

Within the third phase, the potential benefit of web-based information for health related topics will be analyzed. We will provide additional information to lay persons and medical experts via internet, in order to assess the usability of an independent internet search engine making accessible up-to-date, evidence-based and easy to understand information about health topics. To test the potential of information portals for health related topics, the usage of a search engine is evaluated. This engine searches for existing, quality-assured, comprehensible and independent health information across indications. Sources of information accessed by the search engine are reviewed by the Institute for Evidence in Medicine (for Cochrane Germany Foundation) for independence, understandability, being up-to-date and evidence-basis.

Functions of the search engine

- Editorial interface for a community-driven content annotation and curation process
  - Provide a graphical user interface for the editorial process
  - Annotate according to criteria: e.g. correctness, relevance and actuality of contents, evidence-level, user-comprehensibility, others to be defined
  - Implement a community discussion and balloting process
- Search interface
  - Easy patient-centered graphical user-interface for search dialogue and results list
  - Rank results according to criteria
  - Transparent visualization of criteria applied for ranking


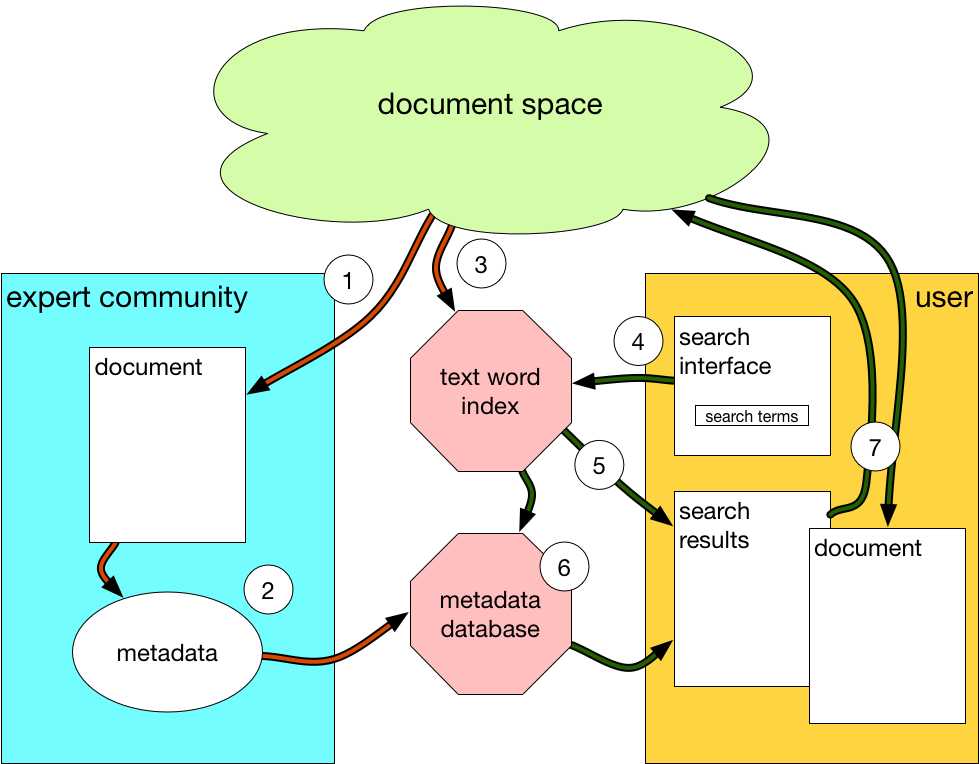
Processes

1. Relevant information is selected and retrieved by an expert community from document space. Document space is a combination of the internet and locally provided databases.
2. Experts annotate the document according to criteria via a graphical user interface. Criteria are e.g. correctness, accuracy, actuality of information, lay comprehensibility of presentation, and the level of evidence. Further information is provided describing the contents. The metadata is stored in a separate database.
3. The text of the web page is text indexed. The normalized index is stored in a text word index.
4. The user formulates his query via a simple search interface. The query term is normalized and searched in the text word index.
5. The links to the documents meeting the user query are retrieved from the text word index and displayed as a list of search results.
6. In parallel, the corresponding metadata on the hits found by the query are retrieved from the metadata database. The search results are sorted (ranking) and displayed according to the metadata. The metadata selection, sorting and presentation options can be configured by the user.
7. The document which has been selected by the user is retrieved from document space via its links and displayed in the browser of the user.

**Development of the search engine**

- The web-interface and search engine will be based on publicly available open-source components.
- The natural language processing (NLP) tool chain for the indexing of web pages and normalization of user search strings will be based on commercially available NLP tools.
- The thesaurus providing access to synonyms and phrases in German will also be based on a commercially available thesaurus.

**Profile of the embedded study on the search machine**

*Research Question:* How easy to understand, navigate and use is a search engine searching information on the internet for health problems other than back pain?

*Population:* 25 general practitioners and patients (without back pain) will be interviewed. ≤ 200 general practitioners and patients participate in an online survey

*Intervention:* Using the search engine for evidence-based, up-to-date, and independent information in plain language for health problems other than back pain

*Outcomes:* (1) Users’ satisfaction with and expectations regarding the search engine assessed by user interviews, (2) acceptance, quality and practicability of the search engine, (3) actual use assessed by specific web-analytics.

Details and procedures of the embedded study

1. Pilot testing of the search engine: The responsible researcher will instruct 25 physicians and citizens interested in health information in the use of the search engine and the study procedure. After giving informed consent, participants use the prototype with specific search queries and a free search and are subsequently asked in semi-structured interviews about acceptance, quality and practicability of the search engine. Afterwards, the interview participants will receive a book voucher. On the basis of these pilot results and published surveys, an online questionnaire on the usability of the search engine will be developed.
2. For evaluating the final version of the search engine, physicians and citizens interested in health information will be contacted and recruited via internet and other channels (physician online networks, support groups). Based on those contacts and the network of the Institute for Evidence in Medicine (for Cochrane Germany Foundation), they will be proactively invited to the search engine and study participation. For further recruitment, they will also be asked to share the link with colleagues and acquaintances.
3. The final version of the search engine will be freely accessible on the internet as a separate portal. However, prior to access, potential users are asked to anonymously fill out a questionnaire on basic data. First, the willingness to complete an online questionnaire (yes / no) after or during the use of the search engine is inquired. If a candidate answers “no”, he can use the search engine nevertheless and is evaluated exclusively by access statistics (numbers and time of access, analysis of query terms). If the candidate answers with “yes”, the following additional basic data will be asked: search as a physician (yes / no), age group (10-year steps), and self-estimated internet affinity. These users are then prompted to participate in the online survey after using the search engine for a certain time or a predetermined number of search queries. It is planned that up to 200 users will participate in the online survey.
4. Outcomes: The online questionnaire asks for the acceptance, quality and practicability of the search engine. Actual usage and search or navigation behaviour is evaluated by web analytics. The users’ search behaviour and the access statistics provide information about the structure of frequently used health information. Further indication areas and preferred user-friendly formats of information processing can be identified for following projects. It will be possible to design studies that will evaluate correlations between the use of cross-indication information portals and general practitioner consultations. In a population-based Dutch observational study involving nearly 1 million patients, a 12% reduction in general practitioner consultations was observed. A survey among NHS Choices users also showed a trend towards reducing consultations.
