# Supplementary material for: Well informed physician-patient communication in consultations on back pain – study protocol of the cluster randomized GAP trial
Source: BMC Fam Pract. 2019 Feb 25;20:33. doi: 10.1186/s12875-019-0925-8 (PMC6388488; doi:10.1186/s12875-019-0925-8)
Supplement: Supplementary file 3 — Characteristics of survey instruments. (DOCX 30 kb) [file 12875_2019_925_MOESM3_ESM.docx]

**GAP trial, additional file 3: Characteristics of survey instruments**

|  | **Used Scales** | **Number of used items** | **Response scale** | **Calculation of score** | **Psychometric properties** |
| --- | --- | --- | --- | --- | --- |
| **Primary outcomes (Survey data)** | | | | | |
| **Quality of the patient-physician communication (patient)** | | | | | |
| Shared Decision-Making Questionnaire (SDM-Q-9) [30] | Complete questionnaire (only one scale) | 9 | 6-point Likert Scale | sum score can be lineally transformed to a scale from 0 to 100 | - good reliability (high internal consistency with a Cronbach’s α = 0.938; high item discrimination indices from 0.685 to 0.826) - good construct validity (one-dimensional structure; 62.4 % variance explained). - medium-sized correlations of a previous version of the SDM-Q-9 with the sub-scales of the Perceived Involvement in Care Scale (PICS) [30], but no convergent validity of the recent version could be established by using the OPTION Scale. [54] |
| Questionnaire on the communication behaviour of physicians (KOVA) [31] | Complete questionnaire   - Patient participation and patient orientation - effective and open communication; - emotionally supportive communication - communication about personal circumstances. | 32 | 6-point Likert Scale | Scale means are transformed to a scale from 0 to 100 | - the scales are reliable (Cronbach’s α between 0.88 and 0.93) - the unidimensionality is satisfactory to very good. |
| P.A.INT-questionnaire [32, 33, 34, 35] | - Satisfaction | 2 | 5-point Likert Scale | Sum or average of scale | - good reliability of the long version of the scales (Cronbach’s α between 0.71 and 0.92) in a rehabilitation setting (with exception of the scales decision making and disturbing factors) |
| **Quality of the patient-physician communication (physician)** | | | | | |
| Shared Decision-Making Questionnaire (SDM-Q-Doc) [37, 38]^a^ | Complete questionnaire (only one scale) | 9 | 6-point Likert Scale | sum score can be lineally transformed to a scale from 0 to 100 | - good reliability (α = 0.88) in a test sample of GPs and medical specialists. - Item difficulties range from 3.52 to 4.34 on a scale from 0 to 5. - Apart from one item, the item discrimination for all indices was higher than 0.4. |
| Questionnaire on the communication behaviour of physicians (KOVA) [31]^a,b^ | - Effective and open communication | 7 | 6-point Likert Scale | Scale means are transformed to a scale from 0 to 100 | -- |
| P.A.INT-questionnaire [32, 33, 34, 35]^a^ | - Empathy - Openness and coherence - Positive regard and appreciation - Contact barriers - Satisfaction | 12 | 5-point Likert Scale | Sum or average of scale | - good reliability of the long version of the scales (Cronbach’s α between 0.71 and 0.92) in a rehabilitation setting (with exception of the scales decision making and disturbing factors) |
| Man-Son-Hing scale. [36]^a^ | N/A | 1 | 5 point scale | N/A | -- |
| **Informedness (patient)** | | | | | |
| Self-reported knowledge questionnaire | Will be developed for the project and checked for understandability and acceptance in a cognitive pretest using one-on-one interviews with eight patients having experience with back pain [39] | | | | |
| Self-reported knowledge global question | Will be developed for the project. | | | | |
| Perceived informedness questionnaire | Will be developed for the project and checked for understandability and acceptance in a cognitive pretest using one-on-one interviews with eight patients having experience with back pain [39] | | | | |
| Perceived informedness global question | Will be developed for the project. | | | | |
|  | **Used Scales** | **Number of used items** | **Response scale** | **Calculation of score** | **Psychometric properties** |
| **Secondary outcomes** | | | | | |
| **Self-reported self-efficacy (patient)** | | | | | |
| Perceived Efficacy in Patient-Physician Interactions (PEPPI)-Questionnaire [40] | Complete questionnaire (only one scale) | 5 | 11-point-Likert scale | Sum score  (50 indicating the highest perceived self-efficacy) | - Two studies reported a good reliability for the English version (Cronbach’s α = 0.82 and 0.83) [40] - Analyses confirmed most hypotheses regarding its construct validity [40]. |
| One Item of Perceived Efficacy in Patient-Physician Interactions (PEPPI)-Questionnaire [40] | N/A | 1 | 11-point-Likert scale | N/A | -- |
| **Health Literacy (patient)** | | | | | |
| Health Literacy Questionnaire (HLQ) [41, 42] | Complete questionnaire   - Feeling understood and supported by healthcare providers - Having sufficient information to manage my health - Actively managing my health - Social support for health - Appraisal of health information - Ability to actively engage with healthcare providers - Navigating the healthcare system - Ability to find good health information - Understanding health information well enough to know what to do | 44 | 4-point Likert- scale (five scales) and 5-point Likert-scale (four scales) | average score for each scale | - nine-factor model of the original version was confirmed for the German version [42] - good reliability of the scales (Cronbach’s α between 0.77 to 0.91) [42] |
| Health literacy global questions | Will be developed for the project. | | | | |
| **Pain intensity (patient)** | | | | | |
| Visual analog scale (VAS) [43] | N/A | 1 | Visual analog scale with a range from 0 to 100 | Score between 0 and 100 | - The reliability of the VAS in assessing acute pain is sufficiently high [43] |
| **Accordance of provided and needed information (physician)** | | | | | |
| Decision Attitude Scale [44] ^c,d^ | - Adequacy of information - Usability of information | 4 | 5-point Likert-scale | Sum or average of scale | - Moderate to good reliability of the scales (r = 0.49 and 0.64) [44] |
| Self-developed item whether content fits expectations | Will be developed for the project. | | | | |
| **Usability (physician)** | | | | | |
| System Usability Scale (SUS) [45]^c,d^ |  | 10 | 5-point Likert-scale | sum score is transformed to a scale from 0 to 100 | - Repeated very good reliability of 0.9 and more [55] - acceptable levels of concurrent validity [55] - Although studies showed that the SUS is bidimensional, these dimensions are of little practical or theoretical interest. Therefore, it is recommended to use the SUS as a unidimensional measure of perceived usability [55]. |
| Self-developed items on usability^d^ | Will be developed for the project. | | | | |
| **Use of portal (physician)** | | | | | |
| Self-developed items on actual use of portal ^d^ | Will be developed for the project. | | | | |

|  | **Used Scales** | **Number of used items** | **Response scale** | **Calculation of score** | **Psychometric properties** |
| --- | --- | --- | --- | --- | --- |
| **Additional assessed variables (Survey data)** | | | | | |
| Back Belief Questionnaire [46] | Complete questionnaire (only one scale) | 14 (9 content and 5 distractor items) | 5-point scale | sum score, is transformed to a scale from 0 to 100 | - Repeated adequate internal consistency (Cronbach’s α was 0.82 [46] and 0.80 [56]) - Acceptable item-total correlation coefficients (>.48) at baseline and follow-up [56] |
| Items regarding sociodemographic data of patients and physicians, internet use and medical data of patients, structural features of the practice | Will be developed for the project. | | | | |

a Instruments will be adapted so that physicians assess their communication behaviour across consultations.

b Instrument will be adapted for assessing the communication from physician’s perspective

c Instrument will be adapted so that the physicians assess the portal

d only intervention group

References:

[30] Scholl I, Kriston L, Härter M. PEF-FB-9 - Fragebogen zur Partizipativen Entscheidungsfindung (revidierte 9-Item Fassung). Klinische Diagnostik und Evaluation. 2009;4(1):46-49.

[31] Farin E, Gramm L, Schmidt E. Taking into account patients’ communication preferences: Development of an instrument and descriptive results in patients with chronic back pain. Patient Education and Counseling. 2012;86(1):41-48.

[32] Dibbelt S, Fleischer C, Schaidhammer M, Greitemann B. Der P.A.INT- Fragebogen zur Kontaktbewertung (P.A.INT-GBB): Ein Instrument zur Erfassung der Qualität der Patienten-Arzt- Interaktion aus zwei Perspektiven. 16. Rehabilitations-wissenschaftliches Kolloquium in Berlin DRV-Schriften. 2007;72.

[33] Fleischer C. Arzt-Patienten-Interaktion als Beitrag zur Qualitätssicherung in Reha-Kliniken [Doctor–patient-interaction as a contribution to quality assurance in rehabilitation clinics]. Diplomarbeit im Fachbereich Humanwissenschaften, Fach Psychologie der Universität Osnabrück [Diplomathesis in the Department of Human sciences, Psychology, University of Osnabrueck]. 2006.

[34] Dibbelt S, Schaidhammer, M, Fleischer C, Greitemann B. Patient-doctor interaction in rehabilitation: The relationship between perceived interaction quality and long-term treatment results. Patient Education and Counseling. 2009;76:328-335.

[35] Dibbelt S, Schaidhammer M, Fleischer C, Greitemann B. Patient-Arzt-Interaktion in der Rehabilitation: Gibt es einen Zusammenhang zwischen wahrgenommener Interaktionsqualität und langfristigen Behandlungsergebnissen? Rehabilitation 2010; 49: 315–325.

[36] Man-Son-Hing M, Laupacis A, O’Connor AM, Biggs J, Drake E, Yetisir E, Hart RG. A Patient Decision Aid Regarding Antithrombotic Therapy for Stroke Prevention in Atrial Fibrillation: A Randomized Controlled Trial. Journal of the American Medical Association. 1999;282:737-742.

[37] Scholl I, Kriston L, Dirmaier J, Buchholz A, Härter M. Development and psychometric properties of the Shared Decision-making Questionnaire – physician version (shared decisionmaking- Q-Doc). Patient Educ Couns. 2012;88(2):284-90.

[38] Scholl I, Kriston L, Dirmaier J, Buchholz A, Härter M. PEF-FB-Doc. Fragebogen zur Partizipativen Entscheidungsfindung (Arztversion). Hamburg: Universitätsklinikum Hamburg-Eppendorf, Institut und Poliklinik für Medizinische Psychologie. 2010.

[40] Maly RC, Frank JC, Marshall GN, DiMatteo MR, Reuben DB. Perceived efficacy in patient-physician interactions (PEPPI): validation of an instrument in older persons. Journal of the American Geriatrics Society. 1998;46(7): 889-894.

[41] Osborne RH, Batterham R, Elsworth GR, Hawkins M, Buchbinder R. The grounded theory, psychometric development and initial validation of the Health Literacy Questionnaire (HLQ). BMC Public Health. 2013;13:658.

[42] Nolte S, Osborne RH, Dwinger S, Elsworth GR, Conrad ML, Rose M, Härter M, Dirmaier J, Zill JM. German translation, cultural adaptation, and validation of the Health Literacy Questionnaire (HLQ). PLOS ONE. 2017;12(2):e0172340.

[43] Bijur PE, Silver W, Gallagher EJ. Reliability of the Visual Analog Scale for Measurement of Acute Pain. Academic Emergency Medicine. 2001;8(12):1153-1157.

[44] Sainfort F, Booske BC. Measuring Post-decision Satisfaction. Medical Decision-making. 2000;20(1): 51-61.

[46] Elfering A, Mannion AF, Jacobshagen N, Tamcan O, Müller U. Beliefs about back pain predict the recovery rate over 52 consecutive weeks. Scand J Work Environ Health. 2009;35(6):437–445.

***Additional references not cited in the main manuscript***

[54] Scholl I, Kriston L, Dirmaier J, Härter M. Comparing the nine-item Shared Decision-Making Questionnaire to the OPTION Scale - an attempt to establish convergent validity. Health Expectations. 2015;18(1):137–150.

[55] Lewis JR, Sauro J. Revisiting the factor structure of the System Usability Scale. Journal of Usability Studies. 2017;12(4):183-192.

[56] Elfering A, Müller U, Salathé C, Tamcan Ö, Mannion A. Pessimistic back beliefs and lack of exercise: a longitudinal risk study in relation to shoulder, neck, and back pain, Psychology, Health & Medicine 2015,20(7), 767-780, doi:10.1080/13548506.2015.1017824.
